# Structural equation analysis of rural communities in South Africa and Nigeria: social determinants of health, nutritional deficiencies, and perceived HIV symptoms

**DOI:** 10.3389/fpubh.2026.1779230

**Published:** 2026-04-09

**Authors:** Mzolisi Payi, Dominic Abaver, Teke Apalata

**Affiliations:** Faculty of Medicine and Health Sciences, Walter Sisulu University, Mthatha, South Africa

**Keywords:** nutritional deficiency, perceived HIV symptoms, rural African health, social determinants of health, structural equation modelling

## Abstract

**Introduction:**

This study examines the pathways through which social determinants of health influence nutritional deficiency and perceived HIV-related symptoms in rural communities across Southwest Nigeria and the Eastern Cape, South Africa. Specifically, it explores how income, healthcare access, and housing quality are associated with both nutritional status and perceptions of HIV-related symptom burden within these underserved populations.

**Methods:**

Using structural equation modelling (SEM), the study analysed survey data from 496 respondents to assess the direct and indirect effects of key social determinants on nutritional deficiency and perceived HIV symptoms. The model incorporated income, access to healthcare, and housing quality as predictor variables. Additionally, one-way ANOVA tests were conducted to explore subgroup differences based on education level and employment status for both nutritional deficiency and perceived HIV-related symptoms.

**Results:**

SEM findings indicate that income and healthcare access each exert significant negative direct effects on nutritional deficiency, highlighting the influence of poverty and limited healthcare utilisation on malnutrition in rural contexts. Housing quality showed a positive but non-significant direct relationship with nutritional status, and its indirect pathways through perceived HIV symptoms were minimal. Perceived HIV-related symptoms emerged as a significant mediator, intensifying the association between nutritional deficiency and social determinants, especially income. Complementary ANOVA results revealed no significant differences in nutritional deficiency across education levels, while employment status demonstrated a significant effect on perceived HIV-related symptom burden. No significant subgroup differences were detected for nutritional deficiency.

**Discussion:**

The findings underscore the intertwined roles of material deprivation, healthcare access, and health perceptions in shaping nutritional vulnerability and perceived HIV symptom burden in rural African communities. The significant mediating effect of perceived HIV symptoms suggests that individuals’ symptom interpretations amplify the impact of social disadvantage on nutritional status. These results offer critical policy insights, emphasising the need for integrated interventions that address both structural deprivation and subjective health vulnerabilities. Such perception-sensitive, multisectoral strategies are essential for improving health outcomes in resource-constrained rural environments in Nigeria and South Africa.

## 1 Introduction

The health of individuals living with HIV is influenced by biomedical interventions, social and structural conditions in which they live (1, 2). In sub-Saharan Africa, where the burden of HIV remains disproportionately high, the connection between poverty, malnutrition, and limited access to healthcare continues to compromise treatment efficacy and quality of life, particularly in rural communities (3). These areas often remain peripheral in mainstream health systems and are characterised by inadequate infrastructure, food insecurity, and deprivation. Clinical research has provided insights into HIV pathophysiology and treatment protocols (4, 5), considerably less attention has been given to how social determinants such as income, housing quality, and access to care interact with nutritional factors to affect individuals’ subjective experiences of HIV-related symptoms.

Perceived symptoms are increasingly recognised as to understanding health among people living with HIV, especially in low-resource areas. These perceptions influence health-seeking behaviour and adherence to antiretroviral therapy (ART) and reflect lived realities that transcend biological explanations (6). Importantly, nutritional deficiency has been shown to contribute to symptom expression and immune suppression, yet its social underpinnings in rural African areas remain insufficiently theorised and empirically carried out (7, 8). How material and social conditions affect both nutrition and symptom perception is therefore essential for designing integrative and locally responsive interventions (9, 10). However, few studies have modelled these interrelations simultaneously, and fewer still have done so using statistical techniques capable of capturing latent, multidimensional constructs (11, 12). However, despite global commitments to addressing health inequities, the persistence of structural disadvantage across rural Africa continues to undermine the social determinants of health (SDH). Income poverty, inadequate housing, and restricted access to healthcare are enduring features of marginalisation and potent predictors of malnutrition, itself a factor in HIV morbidity. These structural conditions worsen nutritional vulnerability, particularly among people living with HIV, for whom dietary adequacy plays a fundamental role in symptom management, immune system regulation, and treatment effectiveness (13, 14). However, much of the existing literature tends to treat these factors in isolation, often privileging biomedical indicators while neglecting the syndemic interactions between social environments, nutritional status, and the lived experience of illness.

To address existing gaps, this study adopts an integrated approach using Structural Equation Modelling (SEM) to examine both direct and mediated links between social conditions and health. SEM enables the analysis of latent variables and structural pathways, capturing how distal social determinants affect nutrition directly and indirectly via perceived HIV symptoms. Existing comparative studies in these areas have largely overlooked the mediating role of perceived symptoms and the extent to which structural determinants influence clinical activities and the internalised experience of disease (15–18). Centring perception within the analytical model, this study responds to growing calls within global health scholarship to move beyond biomedical metrics and to engage more with the subjective dimensions of health and well-being. This study investigates how key social determinants—income, healthcare access, and housing quality—influence nutritional deficiency and perceived HIV symptoms in rural Nigeria and South Africa. Using Structural Equation Modelling (SEM), it examines both direct effects on nutrition and indirect effects mediated by symptom perception. The main objective of this study is to examine the direct and indirect pathways through which key social determinants of health influence nutritional deficiency and perceived HIV-related symptoms among rural populations, using Structural Equation Modelling (SEM). The specific objectives are as follows:

- To assess the direct effects of income, healthcare access, and housing quality on nutritional deficiency among people living with or affected by HIV in rural Nigeria and South Africa.
- To assess the direct effects of income, healthcare access, and housing quality on perceived HIV-related symptoms in the same population.
- To evaluate the mediating role of perceived HIV-related symptoms in the relationship between social determinants and nutritional deficiency.
- To evaluate the mediating role of nutritional deficiency in the relationship between social determinants and perceived HIV-related symptoms.
- To compare patterns of nutritional deficiency and perceived HIV symptomatology across different demographic groups (e.g., education level, employment status) using complementary statistical techniques such as one-way ANOVA.

## 2 Literature review and development of hypotheses

### 2.1 Brief review of literature review

Nutritional deficiency remains a yet under-addressed factor in the health of people living with HIV, particularly in low-resource rural areas (19). Malnutrition weakens immune function and accelerates HIV disease progression; it also intensifies the severity and frequency of HIV-related symptoms, including fatigue, weight loss, and opportunistic infections (20). Empirical evidence from sub-Saharan Africa has consistently shown that micronutrient deficiencies, especially of vitamins A, B-complex, and zinc, are prevalent among HIV-positive individuals and correlate with poorer clinical results (21, 22). These nutritional challenges are compounded by food insecurity, limited dietary diversity, and disrupted agricultural livelihoods, all of which are widespread in rural regions such as those in Nigeria and South Africa (23). Despite the known biological link between nutrition and HIV morbidity, the structural drivers of nutritional vulnerability, including poverty and healthcare access, remain insufficiently examined as part of an integrated understanding of HIV-related health (19).

Social determinants of health (SDH) such as income, housing quality, and access to healthcare are increasingly recognised as foundational drivers of health disparities in HIV (24, 49). These factors affect individuals’ exposure to risk, their capacity to manage illness, and their engagement with care systems. In rural African environments, inadequate infrastructure, unstable income sources, and poor housing conditions are often normalised, exerting chronic stress that undermines both physical health and psychosocial well-being (25). Beyond their material effects, these structural disadvantages also influence how individuals perceive and interpret their symptoms (26), a crucial dimension in understanding health behaviours and treatment adherence. Perceived symptoms, though subjective, are strongly associated with self-rated health, service utilisation, and quality of life (27). Yet, in many perceived HIV studies, symptom perception is either overlooked or treated as a secondary factor (28), despite its potential to serve as both a barometer of suffering and a mediator between structural factors and health states such as nutrition or disease progression.

Although growing attention has been given to HIV-related health, few studies have modelled the interrelationships among social determinants, nutritional status, and perceived symptoms. Existing research often treats these factors in isolation, limiting theoretical insight and policy relevance. The use of Structural Equation Modelling (SEM) in global health is increasing but remains underutilised in rural African contexts, especially in studies involving latent constructs like subjective symptoms and material deprivation. This study addresses that gap by integrating social, nutritional, and perceptual dimensions through SEM to provide a more comprehensive understanding of health inequities among HIV-affected populations in rural Nigeria and South Africa.

### 2.2 Development of hypotheses

The empirical literature consistently shows the importance of social determinants such as income, healthcare access, and housing quality in promoting health performance among people living with HIV (19). However, these relationships are often studied in isolation, with limited attention to how multiple determinants interact to influence nutritional health and symptom perception, particularly in the rural African environment (15–18); this study addresses this gap by modelling both direct and indirect effects using Structural Equation Modelling (SEM), and by incorporating subjective health perceptions, an often overlooked yet component in health behaviour and wellbeing. Studies have shown that lower income is associated with increased nutritional risk due to constrained access to food and healthcare resources (29, 30). Similarly, limited access to healthcare services often delays nutritional assessment and treatment (31), while substandard housing may contribute to environmental exposures that further compromise nutritional health (32). Based on this, the following hypothesis is proposed:

*H1*: Income, access to healthcare, and housing quality will have significant direct effects on nutritional deficiency among people living with or affected by HIV.

Parallel evidence suggests that these same social determinants also impact perceived symptom burden (33). Economic hardship and poor living conditions have been linked to heightened stress, poor ART adherence, and negative self-assessments of health. Limited healthcare access can cause uncertainty or anxiety around HIV symptoms, which may worsen perceived symptom severity (33). Hence, the second hypothesis is formulated:

*H2*: Income, access to healthcare, and housing quality will have significant direct effects on perceived HIV-related symptoms.

Although some studies suggest that nutritional deficiency may be a consequence of poor socioeconomic conditions (34, 35), its role as a mediator in the pathway to symptom perception has not been adequately explored. Poor nutritional status can worsen physical symptoms of HIV, potentially stressing the health impact of social disadvantage (36); this supports the third hypothesis:

*H3*: Perceived HIV-related symptoms will mediate the relationship between social determinants and nutritional deficiency.

Conversely, symptom perception may also influence nutritional abilities (37). Individuals experiencing frequent or intense symptoms may have reduced appetite, energy, or ability to procure and prepare food, ultimately contributing to malnutrition (38); this reciprocal relationship informs the fourth hypothesis:

*H4*: Nutritional deficiency will mediate the relationship between social determinants and perceived HIV-related symptoms.

Finally, while structural pathways are important, demographic factors such as education and employment status may show how individuals experience and report symptoms or nutritional challenges. However, evidence from low-resource rural environments remains inconclusive (39), with some studies suggesting little variation across groups. This study, therefore, also examines subgroup variation as an exploratory aim:

*H5*: There will be significant differences in reported nutritional deficiency and perceived HIV-related symptoms across demographic groups, such as education level and employment status.

## 3 Methodology

### 3.1 Study design

This study adopts a quantitative cross-sectional research design to examine the impact of Social Determinants of Health (SDH) on Perceived HIV Symptoms (hereinafter, HIV) and nutritional deficiencies in selected regions of Nigeria and South Africa. Nutritional deficiencies affect the human immune system, leading to a condition called nutritionally acquired immunodeficiency. Immunodeficiency also affects nutritional deficiency. From a theoretical perspective, human immunodeficiency and nutritional deficiency were necessary to mediate the relationships between social determinants and human immunodeficiency, as well as between social determinants and nutritional deficiency. The mediation models are as follows:

$$\mathrm{nmd}=\lambda_{0}+\lambda_{1}\mathrm{hiv}+\lambda_{2}\mathrm{sd}1+\lambda_{3}\mathrm{sd}4+\lambda_{4}\mathrm{sd}5+\varepsilon$$ (1)

$$\mathrm{hiv}=\theta_{0}+\theta_{1}\mathrm{nmd}+\theta_{2}\mathrm{sd}1+\theta_{3}\mathrm{sd}4+\theta_{4}\mathrm{sd}5+\mu$$ (2)

In Equations 1, 2, NMD is nutritional deficiency, HIV is human immunodeficiency, SD1 is income, SD4 is health care, and SD5 is housing quality and sanitation. The variables are written in lowercase to reflect that they are observed (measured) variables, following standard structural modelling notation, where only latent variables are denoted with capital letters. Although $\lambda_{0}$ and $\theta_{0}$ are constants within the model; they are more accurately interpreted as intercept terms, fixed values that represent the expected level of the dependent variable when all independent variables are equal to zero. Likewise, $\lambda_{\left(i=1,2,3,4\right)}$ = the proportion of the variation in nutritional deficiency that is explained by variations in social health determinants (income, health care, housing quality and sanitation). Also, $\theta_{\left(i=1,2,3,4\right)}$ is the proportion of the variation in HIV that is explained by social determinants (income, health care, housing quality and sanitation). The respective disturbance terms are ε and μ . The study employed the *F* statistic to test for differences in respondents’ perceptions of HIV and nutritional deficiency across education levels (SD2) and employment status (SD3: employed vs. unemployed). The F test was appropriate for comparing group means where more than two groups were involved, as recommended by Armstrong et al. (40).

### 3.2 Operational definition (perceived HIV-related symptoms)

In this study, perceived HIV-related symptoms refer to respondents’ self-reported perception of experiencing symptoms commonly associated with HIV-related illness, captured via a structured questionnaire. This construct reflects subjective symptom perception/symptom burden, not confirmed HIV infection. The perceived HIV-related symptoms variable was measured using four self-report items (5-point Likert scale), with higher scores indicating greater perceived symptom burden. Items were framed as statements about common HIV-related symptoms such as fatigue, unintentional weight loss, and recurrent/opportunistic infections (e.g., ‘I have experienced persistent fatigue/weakness,’ ‘I have experienced unexplained weight loss,’ ‘I have experienced frequent or recurring infections/illness’). Important note: No biomarker or clinical assessment was collected; therefore, this variable is not a measure of HIV status, viral load, CD4 count, or clinical diagnosis.

### 3.3 Conceptual rationale and model specification for reciprocal pathways

Perceived HIV-related symptom burden and nutritional deficiency may plausibly reinforce one another in rural, resource-constrained contexts. First, poorer nutrition can increase fatigue, weight loss, and vulnerability to recurring illness, which may heighten respondents’ perceived symptom burden and overall health-related distress. Second, a higher perceived symptom burden may reduce appetite, energy, and the ability to obtain or prepare food, thereby increasing the risk of nutritional deficiency. This bidirectional logic is consistent with viewing material deprivation, nutritional vulnerability, and lived illness experiences as interacting and mutually reinforcing rather than operating in isolation. Given the cross-sectional design, we do not make strong temporal claims about which process occurs first. Instead, we evaluate reciprocal mechanisms by estimating two complementary recursive mediation SEMs aligned with the study objectives: (i) a model in which nutritional deficiency mediates the association between social determinants of health and perceived HIV-related symptom burden, and (ii) a model in which perceived HIV-related symptom burden mediates the association between social determinants of health and nutritional deficiency. Each model is identified using standard SEM identification procedures for latent-variable models (each construct is measured with multiple observed indicators and scaled by fixing one loading/variance), and model adequacy is supported by the reported diagnostic tests and fit summaries.

### 3.4 Study area

The study was designed to analyse common SDH-related pathways across two comparable rural African environments, rather than to make a simple country-level comparison. The study is set in South-West Nigeria and the Eastern Cape region of South Africa, focusing on Iwo Local Government Area (LGA) in Osun State, Nigeria, and Ntabankulu Local Municipality in the Eastern Cape, South Africa. In Nigeria, Iwo LGA (Agberire, Ologele, and Akinbade villages) is a predominantly rural area characterised by low industrialisation, a heavy reliance on informal trade, and subsistence farming. The area also experiences persistent socioeconomic vulnerability, reflected in limited infrastructure and constrained access to essential services, including healthcare, making it an appropriate context for examining how income, healthcare access, and housing/sanitation conditions shape nutritional deficiency and perceived HIV-related symptoms. In South Africa, Ntabankulu Local Municipality similarly reflects entrenched rural deprivation, with high unemployment and poverty-related constraints on service delivery, supporting its selection as a comparable rural area for the study.

### 3.5 Study population (inclusion and exclusion criteria) and sample size calculation

The study targeted individuals aged 18–65, reflecting the working-age population actively engaged in economic activities and potentially exposed to economic vulnerabilities affecting health. Those under 18, over 65, or unable to provide informed consent were excluded. Sample size was determined using Cochran’s formula to ensure statistical reliability despite uncertain population data (41). The calculated sample sizes—312 for Nigeria and 343 for South Africa—were proportionally distributed across study sites to maintain representativeness (Table 1). A stratified systematic sampling strategy was used in Iwo LGA (Nigeria) and Ntabankulu Municipality (South Africa), where formal household listings were unavailable. Administrative subdivisions (e.g., wards/villages) served as strata. Households were identified via walk-throughs, numbered, and selected systematically using a random start (1–3), followed by every third household (e.g., 2, 5, 8). This ensured randomness and fieldwork feasibility in a rural environment. Field teams accounted for spatial and social patterns to prevent sampling bias. When potential socioeconomic or geographic clustering was detected, additional random checks and alternative paths were introduced to preserve sample integrity. National demographic data from the UN Population Fund (2022) supported the population assumptions used in calculating age-specific sample sizes.

**Table 1:** Summary of the rural environment and adjusted sample sizes.

Summary of the rural environment		
Feature	Nigeria (Iwo LGA)	South Africa (Ntabankulu LM)
Region	Osun State, Southwestern Nigeria	Eastern Cape Province, South Africa
Selected villages	Agberire, Ologele, Akinbade	Saphukanduku, Cedarville, Mpendla, Nhalwane
Population range (Est.)	3,000–9,000 (combined)	5,000+ (combined from two known population villages)
Main livelihood	Small-scale farming	Subsistence agriculture
Languages spoken	Yoruba	isiXhosa
Key challenges	Poor infrastructure and education access	Poverty, service delivery, and unemployment

Summary of sample sizes			
Country	Study area	Population aged 18–65	Calculated minimum sample size
Nigeria	Agberire, Ologele, Akinbade	1,650	312
South Africa	Saphukanduku, Cedarville, Mpendla, Nhalwane	3,185	343

Source: author’s computation.

In Nigeria, 350 questionnaires were distributed, and 208 complete, eligible questionnaires were retained for analysis (usable response rate: 59.4%). In South Africa, 400 questionnaires were distributed, and 288 complete, eligible questionnaires were retained (usable response rate: 72.0%). Where fieldworkers recorded reasons for non-response or exclusion, these were primarily: refusal to participate (e.g., lack of interest or privacy concerns), respondent unavailable at the time of visit (not at home/closed household), time constraints, language/literacy barriers, and incomplete questionnaires that did not meet the study’s minimum completeness criteria. In Table 1, Calculated Minimum Sample Size is the smallest number of participants required for the study, as determined by a sample size formula, to produce statistically reliable results.

### 3.6 Data collection method

Data were collected via structured questionnaires distributed at community centres in Iwo LGA and Ntabankulu Municipality, strategic locations accessible to diverse populations. Trained research assistants ensured informed consent, clarified study details, and supported data collection to improve response rates and data quality. Responses were systematically recorded, anonymised, and securely stored. Data cleaning was performed to resolve inconsistencies, and data management followed strict coding and confidentiality protocols.

### 3.7 Validity and reliability of the instrument

Multiple procedures were used to ensure the questionnaire’s validity and reliability. Content validity was assessed qualitatively through expert review to confirm item relevance, clarity, and adequate coverage of the study constructs before field administration; no statistical content validity index (e.g., CVI/CVR) was computed. A pilot administration was used to refine wording and sequencing and to confirm the instrument’s feasibility. Construct validity was subsequently evaluated using exploratory and confirmatory factor analytic procedures, including convergent and discriminant validity checks, while internal consistency was assessed using composite reliability.

### 3.8 Data handling and statistical analysis

Participant identities were protected through anonymisation, and data were securely stored. The analysis combined descriptive statistics for demographics and key variables with inferential tests, including the White test, equation-level goodness-of-fit tests, the Wald test, and the Likelihood Ratio test, to assess model fit. Structural Equation Modelling with path analysis was used to examine the significance of relationships between social determinants of health (SDH) and health outcomes. Data will be retained for at least 5 years, after which sensitive records will be securely deleted in line with ethical standards.

A questionnaire was classified as a valid response if the respondent met the eligibility criteria (age 18–65 and provided informed consent) and provided complete responses for the items required to compute the key study constructs used in the SEM analysis (income, healthcare access, housing/sanitation, nutritional deficiency, and perceived HIV-related symptoms). Questionnaires missing responses needed to compute one or more construct scores, or those that were substantially incomplete, were excluded during data cleaning. Minor missing values in non-core demographic fields were retained as missing and did not affect inclusion if all construct items were complete. For the ANOVA comparisons, each construct was operationalised as a composite score computed as the mean of its retained items (as listed in Supplementary Table A12), and these analyses are reported for descriptive purposes only.

### 3.9 Ethics approval and permissions

Ethical clearance for this study was obtained from the Walter Sisulu University Health Sciences Research Ethics Committee (WSU HREC; NHREC registration number: REC-120209-020; ethics approval number: WSU HREC 020/2025). Permission to access the study communities and conduct data collection was also obtained from relevant local gatekeepers. In Nigeria, community entry approval was granted by the King (Oba) of Iwo town, and in South Africa, gatekeeper approval was obtained from the Chief of Ntabankulu. Participation was voluntary and based on informed consent; no identifying information was collected, and responses were treated as confidential and used solely for research purposes.

## 4 Results and interpretation

### 4.1 Survey distribution, response rates, and demographic characteristics

Structured questionnaires were administered across seven rural villages, three in Southwest Nigeria and four in the Eastern Cape, South Africa, selected for their shared socio-economic and cultural profiles to ensure analytic comparability. Sampling targets were calculated using Cochran’s formula at a 95% confidence level and 5% margin of error, yielding minimum required sample sizes of 312 for Nigeria and 343 for South Africa, as shown in Supplementary Table A1 in the Appendix section. To mitigate anticipated non-response, 350 and 400 questionnaires were distributed, respectively. A total of 496 valid responses were ultimately collected, 208 from Nigeria and 288 from South Africa. Here, ‘valid responses’ refers to questionnaires from eligible consenting participants with complete responses for the items required to compute the constructs used in the SEM; substantially incomplete questionnaires were excluded during data cleaning. Although the Nigerian sample fell slightly below the target, the combined dataset exceeded the minimum threshold for robust statistical analysis. The comparability of sites and the use of a standardised instrument across all locations supported the decision to conduct a unified analysis.

### 4.2 Descriptive statistics and measurement model evaluation

Supplementary Table A1 outlines the demographic profile of the 496 respondents. Gender distribution was balanced (47.0% male, 53.0% female), supporting representativeness. Most participants (67.9%) held a secondary school certificate, while only 13.4% reported tertiary education (first degree, master’s, or PhD), and 18.8% listed “Other” education, likely vocational or informal, suggesting limited formal education with potential health literacy implications. Occupationally, 48.8% were self-employed, and 34.3% fell under “Others,” indicating a predominantly informal labour force. Formal-sector roles were limited, with 11.1% in healthcare and small fractions in public service, the military, the clergy, and politics.

#### 4.2.1 Descriptive statistics

Supplementary Table A8 provides descriptive statistics for five key variables—income (SD1), healthcare access (SD4), sanitation quality (SD5), nutritional deficiencies (NMD), and HIV prevalence—measured on a 5-point Likert scale (*N* = 496). Mean scores ranged from 2.406 (income) to 3.352 (nutritional deficiencies), indicating limited income and notable perceived nutritional challenges. Healthcare (M = 2.833), sanitation (M = 2.622), and HIV prevalence (M = 2.822) showed moderate levels, reflecting limitations in rural development. Standard deviations (0.842–1.221) indicated moderate variability, with greater dispersion for HIV and nutritional deficiencies, and more uniform responses for income and sanitation.

#### 4.2.2 Measurement model evaluation

##### 4.2.2.1 Tests for sampling adequacy and sphericity

To assess construct validity and the adequacy of the measurement model, exploratory and confirmatory factor analyses were conducted (KMO, Bartlett’s test, AVE, and Fornell-Larcker) as shown in Supplementary Table A1. Factor loadings were subsequently used to compute the Average Variance Extracted (AVE) for each construct as a measure of convergent validity. The Kaiser–Meyer–Olkin (KMO) Measure of Sampling Adequacy, presented in Supplementary Table A2, exceeded the minimum threshold of 0.50, indicating that the sample was adequate for factor analysis. Additionally, Bartlett’s Test of Sphericity yielded a statistically significant result (*χ*^2^ = 231, *p* < 0.001), confirming that the correlation matrix is not an identity matrix and that the dataset meets the necessary assumptions for factor extraction. The AVE values for all constructs exceeded the recommended benchmark of 0.50, indicating that the latent variables account for more than half of the variance in their observed indicators.

##### 4.2.2.2 Validity test result

Supplementary Table A3 shows factor loadings reflecting the association between observed items and their latent constructs. Income (0.722) and HIV (0.693) had strong loadings, while Healthcare (0.538), Housing and Sanitation (0.567), and Nutritional Deficiency (0.513) showed moderate alignment. Although higher loadings support item representation, construct validity also depends on conceptual clarity and interpretation. Discriminant validity, assessed via the Fornell–Larcker criterion, was confirmed: the square roots of AVEs (Supplementary Table A4) exceeded inter-construct correlations, indicating each construct was more strongly related to its own indicators than to others. Standardised factor loadings for all indicators, together with item wording and response format, are reported in Supplementary Table A12.

##### 4.2.2.3 Composite reliability

Supplementary Table A5 presents the composite reliability. Since the factor loadings were not homogeneous, the study employed composite reliability (CR) to assess internal consistency, as this metric is particularly appropriate when factor loadings vary across indicators. Unlike Cronbach’s alpha, which assumes equal loadings, composite reliability accounts for the varying contributions of indicators to the latent construct, providing a more accurate assessment of reliability under these conditions. All composite reliability coefficients exceeded the recommended threshold of 0.7, indicating strong internal consistency and further supporting the robustness of the measurement model.

##### 4.2.2.4 Collinearity diagnostics

Collinearity diagnostics using the Variance Inflation Factor (VIF) and Tolerance statistics (Supplementary Table A6) were conducted for models with NMD and HIV as dependent variables. For the NMD model, VIF values ranged from 1.032 to 1.313, and Tolerance values ranged from 0.762 to 0.969, indicating no multicollinearity issues. Similarly, in the HIV model, VIFs ranged from 1.034 (NMD) to 1.357 (SD4), with Tolerance values between 0.737 and 0.967—all within acceptable limits. These results confirm that the predictors are sufficiently independent, ensuring stable estimates and enhancing model robustness and interpretability.

##### 4.2.2.5 Equation-level goodness of fit

Supplementary Table A7 summarises equation-level goodness-of-fit statistics for models assessing how social determinants of health (SDH) affect HIV and nutritional deficiencies (NMD). Key indicators include total, explained, and residual variance, along with R-squared, model correlation (mc), and mc-squared. The initial HIV model explained 60% of the variance (*R*^2^ = 0.600; mc = 0.775), while the NMD model accounted for 42.4% (*R*^2^ = 0.424; mc = 0.651). The system-level *R*^2^ was 0.515, surpassing the 0.50 benchmark common in public health research. Following model refinement, the HIV model showed improved fit with *R*^2^ = 0.553 and mc = 0.743. The NMD model results remained unchanged (*R*^2^ = 0.424; mc = 0.651). The revised overall weighted *R*^2^ was 0.491, still indicating a good explanatory performance across the system.

### 4.3 Results of the inferential statistics

**Figure 1:**
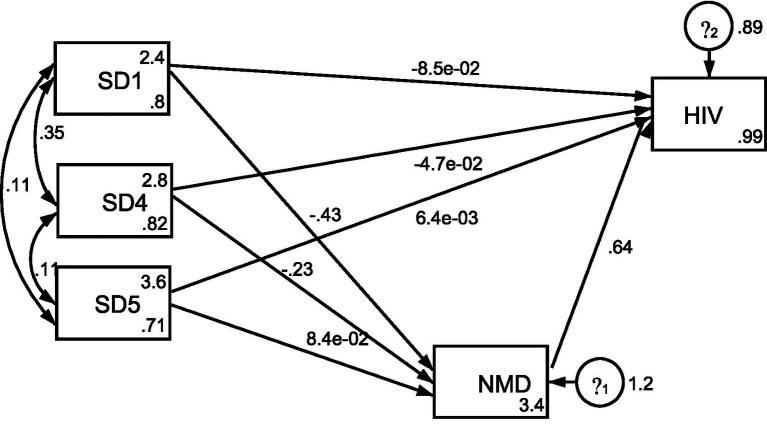
Structural equation model path diagram of SDH and HIV.

Table 2 presents the structural equation model examining the relationship between social determinants of health and HIV. As shown in the path diagram (Figure 1), income and healthcare access demonstrated significant negative associations with nutritional deficiency, with both coefficients achieving statistical significance (*p* < 0.05). This suggests that higher income and improved healthcare access reduce the likelihood of nutritional deficiency, while lower income and limited healthcare increase nutritional vulnerability, findings consistent with prior research linking poverty and constrained access to food and care with higher nutritional risk (29–31). In contrast, although housing quality and sanitation showed a positive association with nutritional deficiency, the relationship was not statistically significant, aligning with existing work suggesting that the impact of substandard housing on nutrition may be indirect or dependent (32).

**Table 2:** Structural equation model of the SDH and HIV.

Variable	Coef.	Std. err.	*z*	*p* > |z|	[95% conf. interval]	
Direct effects						
*NMD*						
SD1	−0.4300692	0.0624028	−6.89	0.000	−0.5523763	−0.307762
SD4	−0.2294565	0.0615971	3.73	0.000	−0.4087284	−0.0501847
SD5	0.0841502	0.0607117	1.39	0.166	−0.0348426	0.2031429
HIV						
NMD	0.6422774	0.0380975	16.86	0.000	0 0.5676077	0.7169472
SD1	−0.085489	0.0552198	−1.55	0.122	−0.1937179	0.0227399
SD4	−0.0474812	0.0527812	−0.90	0.368	0.368000	0.055968
SD5	0.0064413	0.0514041	0.13	0.900	−0.0943089	0.1071915
Indirect effects						
*Structural NMD*						
SD1	0 (no path)					
SD4	0 (no path)					
SD5	0 (no path)					
HIV						
NMD	0 (no path)					
SD1	−0.2762237	0.0432996	−6.38	0.000	−0.3610893	−0.1913581
SD4	−0.1473748	0.0405167	−3.64	0.000	0.0679634	0.2267861
SD5	0.0540477	0.0391253	1.38	0.167	−0.0226365	0 0.130732

SD1, income; SD2, education level; SD3, employment status; SD4, healthcare access; SD5, housing quality and sanitation; NMD, nutritional deficiency; HIV, perceived HIV-related symptoms (human immunodeficiency).

Source: author’s computation.

The model also revealed that nutritional deficiency had a significant positive relationship with perceived HIV-related symptoms, indicating that higher levels of nutritional deficiency are associated with increased susceptibility to immunodeficiency. This finding aligns with evidence that poor nutritional status can worsen HIV symptom severity and contribute to immune system decline (34, 36). However, income, housing quality, and sanitation did not demonstrate significant direct effects on HIV-related symptoms. This suggests that, within the studied rural communities of Nigeria and South Africa, these structural determinants may influence HIV vulnerability more indirectly, potentially through mediators like nutrition rather than exerting strong direct effects, as reflected in similar findings on the attenuated roles of structural determinants in symptom perception (33).

Notably, the analysis of indirect effects, specifically the pathways from social determinants to human immunodeficiency with nutritional deficiency as a mediating variable, revealed that both income and healthcare had statistically significant negative relationships with human immunodeficiency (*p* < 0.05); this means that increased income and better healthcare indirectly reduce the risk of immunodeficiency by lowering nutritional deficiency levels. Importantly, although the direct effects of income and healthcare on immunodeficiency were statistically insignificant, their indirect effects became significant through the mediating role of nutritional deficiency, supporting full mediation. Implies that nutritional deficiency fully explains the pathway linking both income and healthcare to human immunodeficiency.

**Figure 2:**
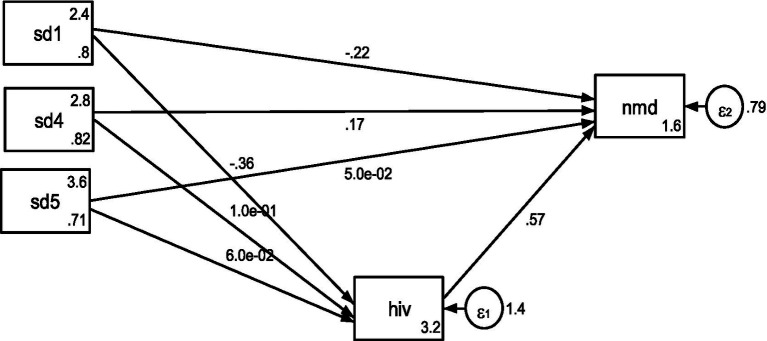
Structural equation model path diagram of SDH and NMD.

The path diagram analysis of the structural equation model examining the direct effects of social determinants of health on nutritional deficiency (Table 3; Figure 2) revealed notable patterns. The coefficient for income was negative and statistically significant (*p* < 0.05), indicating that individuals with higher income are less likely to experience nutritional deficiencies, whereas lower income is associated with increased risk. This supports the established link between poverty and nutritional vulnerability observed in prior studies, where limited income constrains access to adequate food and health resources (29, 30). Conversely, while healthcare access and housing quality/sanitation were positively associated with nutritional deficiency, these relationships were statistically insignificant in the direct model, suggesting no strong direct effects, an outcome that partially aligns with previous evidence indicating that housing-related health risks may be more indirect or dependent (32).

**Table 3:** Structural equation model of the SDH and NMD.

Variable	Coef.	Std. err.	*z*	*p* > |z|	[95% Conf. interval]	
Direct effects						
*HIV*						
SD1	−0.3617127	0.0662357	−5.46	0.000	−0.4915324	−0.231893
SD4	0.0998935	0.0653806	1.53	0.127	−0.0282501	0 0.2280372
SD5	0.0604891	0.0644408	0.94	0.348	−0.0658126	0.1867907
NMD						
HIV	0.5700928	0.0338158	16.86	0.000	0.5038151	0.6363705
SD1	−0.2238593	0.051165	−4.38	0.000	−0.324141	−0.1235777
SD4	0.1725079	0.0491563	3.51	0.000	0.0761634	0.2688525
SD5	0.0496658	0.0483784	1.03	0.305	−0.0451542	0.1444857
Indirect effects						
*Structural HIV*						
SD1	0 (no path)					
SD4	0 (no path)					
SD5	0 (no path)					
NMD						
HIV	0 (no path)					
SD1	−0.2062098	0.0396922	−5.20	0.000	−0.2840051	−0.1284146
SD4	0.0569486	0.0374258	1.52	0.128	−0.0164046	0.1303018
SD5	0.0344844	0.0367941	0.94	0.349	−0.0376308	0.1065996

SD1, income; SD2, education level; SD3, employment status; SD4, healthcare access; SD5, housing quality and sanitation; NMD, nutritional deficiency; HIV, perceived HIV-related symptoms (human immunodeficiency).

Source: author’s computation.

Further analysis revealed that human immunodeficiency exhibited a positive, statistically significant relationship with nutritional deficiency, suggesting that greater immunodeficiency is associated with worse nutritional outcomes. This supports literature linking poor nutritional status with weakened immune function and heightened HIV symptom burden (35, 36). Additionally, the significant negative relationship between income and nutritional deficiency persisted in the extended model, further affirming the protective role of economic resources. Interestingly, healthcare access showed a positive and statistically significant association with nutritional deficiency, an initial result that may reflect the economic strain of healthcare costs in resource-limited areas, where financial outlays for medical care can reduce the ability to meet basic nutritional needs. This aligns with prior findings reporting the trade-offs faced by low-income populations facing health expenses (31).

Regarding indirect effects, the mediation analysis identified human immunodeficiency as a partial mediator in the relationship between income and nutritional deficiency. The negative association between income and nutritional deficiency remained significant even when accounting for immunodeficiency, indicating that income affects nutrition both directly and through its influence on HIV-related vulnerability. In contrast, healthcare, despite its significant direct effect, showed an insignificant indirect relationship via immunodeficiency, suggesting a possible moderating rather than mediating influence. Taken together, these findings support a partial mediation framework in which income maintains a protective role, while the influence of healthcare and housing appears to operate through more pathways.

### 4.4 Analysis of education and employment on nutritional deficiencies and HIV

To assess whether perceptions of HIV varied across demographic groups, a one-way ANOVA was conducted (Table 4). The analysis revealed no significant differences by education level (SD2), with an *F*-statistic of 0.417 and a *p*-value of 0.797. In contrast, employment status (SD3) showed a statistically significant effect, with an *F*-statistic of 10.511 and a *p*-value below 0.01, indicating meaningful variation in HIV-related perceptions between employed and unemployed respondents. These results suggest that employment status, more than formal education, influences how individuals perceive HIV, likely due to the heightened social and economic vulnerabilities experienced by unemployed individuals in rural communities. This finding is consistent with prior research noting that socioeconomic instability and limited occupational engagement can exacerbate stress, reduce ART adherence, and intensify perceived symptom burden among people living with HIV (33).

**Table 4:** ANOVA result for education/employment.

Factor	*F*-statistic	*p*-value	Significance
ANOVA result for education/employment and HIV			
*ANOVA result statistic for HIV model*			
Education (SD2)	0.417	0.797	Not significant
Employment status (SD3)	10.511	<0.01	significant
ANOVA result for education/employment and NMD			
*ANOVA result statistic for NMD model*			
Education (SD2)	1.228	0.298	Not significant
Employment status (SD3)	0.174	0.840	Not significant

SD2, education level; SD3, employment status.

Source: author’s computation.

All items were measured on a 5-point Likert scale (1 = Strongly disagree, 5 = Strongly agree). AVE, average variance extracted; CR, composite reliability.

Source: authors’ computation.

Similarly, the study assessed whether perceptions of nutritional deficiency differed by education and employment status using the same method (Supplementary Table A9). Here, no statistically significant differences were observed: the *F*-statistic was 1.228 (*p* = 0.298) for education and 0.174 (*p* = 0.840) for employment. These results indicate a uniformity in perceptions of nutritional deficiency across demographic subgroups, which may reflect the shared material deprivation and food insecurity often characteristic of rural communities. This aligns with findings from Westbury et al. (39), who noted minimal perceptual differences among structurally marginalised populations, in which access to nutrition tends to be constrained at the community level, regardless of educational attainment or employment status.

### 4.5 Model diagnostic tests

A series of diagnostic tests confirmed the adequacy, robustness, and validity of the structural equation model, as detailed in the appendices. The Wald test (Supplementary Table A9) showed joint predictor significance for both HIV (*χ*^2^ = 30.58, df = 3, *p* < 0.001) and nutritional deficiency (*χ*^2^ = 361.66, df = 4, *p* < 0.001) models. The Likelihood Ratio test (Supplementary Table A10) supported model fit, with the fitted vs. saturated model yielding *χ*^2^ = 0.000 (df = 0), and the null vs. saturated model yielding *χ*^2^ = 300.785 (df = 7, *p* < 0.001). White’s test (Supplementary Table A11) found no heteroskedasticity in the residuals for both the SEM (*χ*^2^ = 14.47, df = 14, *p* = 0.4151) and the HIV model (*χ*^2^ = 13.33, df = 9, *p* = 0.1483). Cameron and Trivedi’s IM decomposition confirmed this with non-significant heteroskedasticity, skewness, and kurtosis components. Overall IM statistics further supported model adequacy for SEM (*χ*^2^ = 18.15, df = 19, *p* = 0.5121) and HIV model (*χ*^2^ = 16.61, df = 13, *p* = 0.2177).

## 5 Conclusion

This study was motivated by the persistent gaps in understanding how social and structural conditions affect health among people living with or affected by HIV in rural African communities. Despite extensive studies on the biomedical dimensions of HIV, the synergistic roles of material deprivation, nutritional deficiency, and subjective health experiences remain underexplored, particularly within underserved rural populations. In response, this study aimed to examine the direct and indirect effects of key social determinants, income, healthcare access, and housing quality on nutritional deficiency and perceived HIV-related symptoms. Employing Structural Equation Modelling (SEM), we tested a bidirectional mediation framework using data from rural communities in Southwest Nigeria and the Eastern Cape of South Africa, thereby allowing the simultaneous estimation of latent relationships and providing a better understanding of how social disadvantage translates into health vulnerability.

The findings reveal how structural disadvantage translates into both physical and perceived health burdens. Income and access to healthcare exhibited significant negative direct effects on nutritional deficiency, indicating the role of poverty and service inaccessibility in driving undernutrition among HIV-affected individuals. While housing quality showed a non-significant association, perceived HIV-related symptoms emerged as a crucial mediator, strengthening the pathway between social deprivation and nutritional impact, particularly in relation to income; this highlights how lived experiences of illness, influenced by both physiological and psychosocial stressors, can worsen nutritional vulnerability in ways that are not captured by clinical measures only. ANOVA results indicate no significant variation by education, but a significant difference by employment status in perceived HIV-related symptom burden, while nutritional deficiency shows no significant differences by education or employment status. The results call for the design of integrated, perception-sensitive public health interventions that address both material deprivation and subjective health experiences within rural African communities.

The study’s results point to an urgent need for structural interventions that go beyond biomedical responses to HIV and address the social realities impacting health in rural environments. The significant effects of income and healthcare access on nutritional deficiency signal that material deprivation continues to be a primary constraint on the well-being of people living with or affected by HIV. Policies that improve rural livelihoods, reduce economic vulnerability, and ensure access to affordable, quality healthcare are likely to yield nutritional benefits and improvements in symptom management and quality of life. Strengthening rural health infrastructure, integrating nutritional services into HIV care, and expanding social protection measures such as conditional cash transfers or food support programs could help break the interlinked cycles of poverty, malnutrition, and illness.

Importantly, the role of perceived HIV symptoms as a mediator stressed the need to incorporate subjective health experiences into policy design. Symptom perception is a reflection of biological health and a product of psychosocial stress, access to care. Incorporating perception-sensitive approaches into HIV programs through culturally grounded health education, psychosocial counselling, and patient-reported outcome tracking could enhance engagement with care and support more responsive interventions. The absence of significant demographic variation across education and employment groups further implies that such strategies must be universally applied across rural populations, rather than narrowly targeted. Taken together, the findings argue for an equity-oriented policy framework that addresses both the material and perceptual dimensions of health vulnerability in underserved communities.

This study makes a significant contribution to the understanding of how structural and perceptual dimensions of health are connected in rural areas burdened by HIV. However, this study may be limited by its cross-sectional design, which precludes causal inference regarding the relationships among social determinants, nutritional status, and perceived HIV symptoms. Additionally, reliance on self-reported data for symptom perception and some social indicators may introduce reporting bias. Future research should employ longitudinal designs to capture temporal dynamics and better establish causal pathways.

## Data Availability

The data presented in the study are available from https://figshare.com/s/72f7226e9bc00e434ebc. Further inquiries can be directed to the corresponding author/s.
