# Supplementary material for: Structural equation analysis of rural communities in South Africa and Nigeria: social determinants of health, nutritional deficiencies, and perceived HIV symptoms
Source: Front Public Health. 2026 Apr 9;14:1779230. doi: 10.3389/fpubh.2026.1779230 (PMC13102834; doi:10.3389/fpubh.2026.1779230)
Supplement: Supplementary file 1 [file Supplementary_file_1.docx]

**Appendix**

**Table A1: Demographic Characteristics**

| **Variable** | **Category** | **Frequency** | **Percentage (%)** |
| --- | --- | --- | --- |
| Gender | Male | 233 | 47.0 |
|  | Female | 263 | 53.0 |
|  | **Total** | **496** | **100.0** |
| Highest Educational Qualification | School Certificate | 337 | 67.9 |
|  | First Degree | 32 | 6.5 |
|  | Master's Degree | 32 | 6.5 |
|  | Ph.D. | 2 | 0.4 |
|  | Others | 93 | 18.8 |
|  | **Total** | **496** | **100.0** |
| Occupation | Healthcare Practitioner | 55 | 11.1 |
|  | Self-Employed | 242 | 48.8 |
|  | Public Servant | 9 | 1.8 |
|  | Armed Forces | 4 | 0.8 |
|  | Clergy | 3 | 0.6 |
|  | Politician | 13 | 2.6 |
|  | Others | 170 | 34.3 |
|  | **Total** | **496** | **100.0** |

Source: Author’s Computation

**Table A2:** **Tests for Sampling Adequacy and Sphericity**

| Tests for Sampling Adequacy and Sphericity | |
| --- | --- |
| Kaiser Meyer Olkin Measure of Sampling Adequacy | 0.8320 |
| Bartlett’s Test for Approximate Chi-Square | 0.0043 |
| Sphericity Degree of Freedom | 231 |
| Significance | 0.000 |

Source: Author’s Computation

**Table A3:** **Validity Test Result**

| Validity Result | | | |  |
| --- | --- | --- | --- | --- |
|  | | | Convergent Validity |  |
| Income (SD1) | 2.890/4 | = | 0.722 |  |
|  |  |  |  |  |
| Healthcare ((SD4) | 2.151/4 | = | 0.538 |  |
|  |  |  |  |  |
| Housing Quality and Sanitation (SD5) | 3.402/6 | = | 0.567 |  |
|  |  |  |  |  |
| HIV | 2.770/4 | = | 0.693 |  |
|  |  |  |  |  |
| Nutritional Deficiency (NMD) | 2.053/4 | = | 0.513 |  |
|  |  |  |  |  |

Source: Author’s Computation

**Table A4:** **Divergent Validity**

| Divergent Validity | | | | | |
| --- | --- | --- | --- | --- | --- |
|  | SD1 | SD4 | SD5 | HIV | NMD |
| SD1 | **0.850** |  |  |  |  |
| SD4 | 0.429 | **0.733** |  |  |  |
| SD5 | 0.149 | 0.138 | **0.752** |  |  |
| HIV | -0.228 | -0.034 | 0.013 | **0.832** |  |
| NMD | -0.244 | -0.044 | 0.038 | 0.630 | **0.716** |

Note: SD1 = Income; SD2 = Education level; SD3 = Employment status; SD4 = Healthcare access; SD5 = Housing quality and sanitation; NMD = Nutritional deficiency; HIV = Perceived HIV-related symptoms (human immunodeficiency)

Source: Author’s Computation

**Table A5** **Composite Reliability**

Composite Reliability

SD1 $\frac{{2.89}^{2}}{{2.89}^{2}+1.849}$ = 0.819

SD4 $\frac{{2.995}^{2}}{{2.995}^{2}+ 1.73}$ = 0.838

SD5 $\frac{{4.057}^{2}}{{4.057}^{2}+3.23}$ = 0.836

NMD $\frac{{3.08}^{2}}{{3.08}^{2}+ 1.62.}$ = 0.854

HIV $\frac{{2.858}^{2}}{{2.858}^{2}+ 1.947}$ = 0.808

Note: SD1 = Income; SD2 = Education level; SD3 = Employment status; SD4 = Healthcare access; SD5 = Housing quality and sanitation; NMD = Nutritional deficiency; HIV = Perceived HIV-related symptoms (human immunodeficiency)

Source: Author’s Computation

**Table A6:**  **Collinearity Diagnostics (with NMD as dependent variable)**

| Collinearity Diagnostics (with NMD as dependent variable) | | |
| --- | --- | --- |
| Variable | Tolerance | VIF |
| HIV | 0.941 | 1.062 |
| SD 1 | 0.762 | 1.313 |
| SD4 | 0.807 | 1.240 |
| SD5 | 0.969 | 1.032 |
| Collinearity Diagnostics (with HIV as dependent variable) | | |
| Variable | Tolerance | VIF |
| SD 1 | 0.909 | 1.100 |
| SD4 | 0.737 | 1.357 |
| SD5 | 0.788 | 1.269 |
| NMD | 0.967 | 1.034 |

Note: SD1 = Income; SD2 = Education level; SD3 = Employment status; SD4 = Healthcare access; SD5 = Housing quality and sanitation; NMD = Nutritional deficiency; HIV = Perceived HIV-related symptoms (human immunodeficiency)

Source: Author’s Computation

**Table A7:** **Equation-level goodness of fit for the Model of the SDH and HIV**

| Equation-level goodness of fit for the Model of the SDH and HIV | | | | | | |
| --- | --- | --- | --- | --- | --- | --- |
| Depvar | Total Variance | Residual Variance | Fitted Variance | R-squared | mc | mc-squared |
| HIV | 1.490057 | 0.5960 | 0.8940 | 0.600 | 0.7746 | 0.600 |
| NMD | 1.369426 | 0.7893 | 0.5801 | 0.424 | 0.6507 | 0.424 |
| Overall |  |  |  | 0.515 |  |  |
| Equation-level goodness of fit for the Model of the SDH and NMD | | | | | | |
| Depvar | Total Variance | Residual Variance | Fitted Variance | R-squared | mc | mc-squared |
| HIV | 1.490057 | 0.6666 | 0.8235 | 0.5525 | 0.7433 | 0.5525 |
| NMD | 1.369426 | 0.7893 | 0.5802 | 0.4237 | 0.6509 | 0.4237 |
| Overall |  |  |  | 0.49091 |  |  |

Note: NMD = Nutritional deficiency; HIV = Perceived HIV-related symptoms (human immunodeficiency)

Source: Author’s Computation

| **Variable** | **Mean** | **Standard Deviation** | **Minimum** | **Maximum** | **Valid N** | **Skewness** | **Kurtosis** |
| --- | --- | --- | --- | --- | --- | --- | --- |
| SD1 (Income) | 2.406 | 0.898 | 1.000 | 5.000 | 496 | 0.350 | -0.500 |
| SD4 (Healthcare Access) | 2.833 | 0.907 | 1.000 | 5.000 | 496 | 0.100 | -0.600 |
| SD5 (Quality of Sanitation) | 2.622 | 0.842 | 1.000 | 5.000 | 496 | 0.250 | -0.450 |
| NMD (Nutritional Deficiencies) | 3.352 | 1.171 | 1.000 | 5.000 | 496 | -0.200 | -0.750 |
| HIV | 2.822 | 1.221 | 1.000 | 5.000 | 496 | 0.050 | -0.800 |

**Table A8 Descriptive Statistics**

Source: Author’s Computation

**Table A9 Wald Test Result**

| **Wald tests for equations** | | | |
| --- | --- | --- | --- |
| observed | chi2 | df | p |
| HIV | 30.58 | 3 | 0.0000 |
| NMD | 361.66 | 4 | 0.0000 |

Source: Author’s Computation

**Table A10 Likelihood Ratio Test**

| **Likelihood Ratio Test** | | |
| --- | --- | --- |
| Fit statistic | Value | Description |
| Likelihood ratio |  |  |
| chi2_ms(0) | 0.000 | 0.000 model vs. saturated |
| p > chi2 |  |  |
| chi2_bs(7) | 300.785 | baseline vs. saturated |
| p > chi2 | 0.000 |  |

Source: Author’s Computation

**Table A11 White's Test Result**

| White's Test Result for NMD as Dependent Variable | | | |
| --- | --- | --- | --- |
| White's test for Ho: homoskedasticity | | | |
| Against Ha: unrestricted heteroskedasticity | | | |
| chi2(14) = 14.47 | | | |
| Prob > chi2 = 0.4151 | | | |
| Cameron & Trivedi's decomposition of IM-test | | | |
| Source | chi2 | df | p |
| Heteroskedasticity | 14.47 | 14 | 0.4151 |
| Skewness | 2.51 | 4 | 0.6431 |
| Kurtosis | 1.17 | 1 | 0.2787 |
| Total | 18.15 | 19 | 0.5121 |
| White's Test Result for HIV as Dependent Variable | | | |
| White's test for Ho: homoskedasticity | | | |
| Against Ha: unrestricted heteroskedasticity | | | |
| chi2(9) = 13.33 | | | |
| Prob > chi2 = 0.1483 | | | |
| Cameron & Trivedi's decomposition of IM-test | | | |
| Source | chi2 | df | p |
| Heteroskedasticity | 13.33 | 9 | 0.1483 |
| Skewness | 2.11 | 4 | 0.5501 |
| Kurtosis | 1.17 | 1 | 0.2787 |
| Total | 16.61 | 13 | 0.2177 |

Source: Author’s Computation

**Table A12 Measurement items, response format, and standardised loadings (λ)**

| Construct (Latent Variable) | Item Code | Questionnaire Item Wording | Standardised Loading (λ) |
| --- | --- | --- | --- |
| Income (SD1) | SD1_1 | I earn income regularly. | 0.79 |
|  | SD1_2 | My income is adequate for my needs. | 0.84 |
|  | SD1_3 | I am able to pay my bills with my regular income. | 0.87 |
|  | SD1_4 | I am able to save from my income. | 0.81 |
| Construct‑level metrics: AVE = 0.686; CR = 0.897 | | | |
| Healthcare Access (SD4) | SD4_1 | I am enrolled in a health insurance scheme. | 0.62 |
|  | SD4_2 | I have access to clinical preventive services. | 0.75 |
|  | SD4_3 | I can afford healthcare services. | 0.7 |
|  | SD4_4 | I have access to good healthcare facilities. | 0.78 |
| Construct‑level metrics: AVE = 0.511; CR = 0.806 | | | |
| Housing Quality and Sanitation (SD5) | SD5_1 | The rooms in my home are not overcrowded. | 0.74 |
|  | SD5_2 | The rooms in my home are well‑ventilated. | 0.71 |
|  | SD5_3 | The surroundings of my home are clean. | 0.67 |
|  | SD5_4 | I have access to safe drinking water. | 0.76 |
|  | SD5_5 | My home is adequately lit. | 0.69 |
|  | SD5_6 | The sanitation facilities in my home are adequate. | 0.6 |
| Construct‑level metrics: AVE = 0.486; CR = 0.849 | | | |
| Perceived HIV‑Related Symptoms (PHRS) | PHRS_1 | I frequently experience pneumonia-like symptoms. | 0.8 |
|  | PHRS_2 | I sometimes experience infections in different parts of my body. | 0.68 |
|  | PHRS_3 | I sometimes suffer from low blood‑platelet-related symptoms. | 0.73 |
|  | PHRS_4 | I experience digestive problems such as loss of appetite or diarrhoea. | 0.77 |
| Construct‑level metrics: AVE = 0.557; CR = 0.834 | | | |
| Nutritional Deficiency (NMD) | NMD_1 | I frequently experience fatigue. | 0.83 |
|  | NMD_2 | I often feel dizzy. | 0.74 |
|  | NMD_3 | I experience weakness in my muscles. | 0.79 |
|  | NMD_4 | I sometimes experience weight loss. | 0.71 |
| Construct‑level metrics: AVE = 0.591; CR = 0.852 | | | |

All items were measured on a 5‑point Likert scale (1 = Strongly disagree, 5 = Strongly agree). AVE = average variance extracted; CR = composite reliability.

Source: Authors’ Computation
